# Comparative adsorption performance of bare and chitosan-coated La-Zn-Fe spinel/perovskite nanocomposites for efficient dye removal

**DOI:** 10.1038/s41598-026-55233-1

**Published:** 2026-06-24

**Authors:** Ehab A. Okba, Hend Mansour, Shaban Y. Shaban

**Affiliations:** 1 Chemistry Department, Faculty of Science, Tanta University, Tanta, 31527 Egypt; 2 Chemistry Department, Faculty of Science, Kafrelsheikh University, Kafrelsheikh, 33516 Egypt

**Keywords:** Metal ferrite, Cationic and anionic dyes, Adsorption, Wastewater treatment, Chemistry, Environmental sciences, Materials science, Nanoscience and technology

## Abstract

Effective and affordable treatment of dye-contaminated wastewater remains essential for environmental protection. This study reports the synthesis of La_0.5_Zn_0.5_Fe_2_O_4_ (LZNF) nanoparticles via coprecipitation and their in situ polymerization with Chitosan to form LZNF/CS nanocomposites. The materials were characterized using EDX, FT-IR, VSM, XRD, TEM, SEM, and BET analyses. XRD confirmed nanocrystalline cubic spinel La_0.5_Zn_0.5_Fe₂O₄ as the main phase, with orthorhombic perovskite LaFeO₃ as a secondary component. LZNF showed high adsorption efficiency toward the anionic dyes indigo carmine (IC) and Acid Blue 25, achieving removals of 97.7% and 92%, respectively, while LZNF/CS achieved 92% and 69% under acidic conditions. Adsorption of both dyes followed a pseudo-second-order kinetic model and was spontaneous and exothermic. IC and AB 25 adsorption on LZNF fitted the Langmuir model, indicating monolayer adsorption, whereas adsorption on LZNF/CS followed the Freundlich model, suggesting multilayer uptake due to Chitosan modification. Electrostatic interactions dominated the adsorption mechanism, with hydrogen bonding and electrostatic interactions contributing secondarily. Both materials retained strong performance over five regeneration cycles, supporting their potential as efficient adsorbents for wastewater purification.

## Introduction

Water pollution has become a pressing global issue due to the rapid increase in industrialisation and urbanisation, which discharge large amounts of untreated effluents into natural water bodies. Among the various contaminants, synthetic dyes are particularly hazardous due to their complex aromatic structures, high stability, and resistance to biodegradation, leading to severe ecological and health problems^1^. These pollutants not only reduce light penetration in aquatic systems, disrupting photosynthesis, but also release toxic, mutagenic, and carcinogenic by-products during degradation.

Several techniques, including membrane filtration, coagulation, flocculation, reverse osmosis, and adsorption, have been used to remove colour from contaminated water systems^2–5^.. Adsorption is considered a simple, cost-effective, safe, and efficient technique for dye removal. Various adsorbents have been employed for this purpose, including bentonite^6^, clay minerals^7^, and adsorbents derived from agricultural waste^8^.

An efficient approach for dye removal involves the use of magnetic materials, particularly metal ferrites. Their magnetic properties make metal ferrites highly effective in adsorbing and removing various dye compounds from wastewater. These materials have unique characteristics, such as a large surface area, strong magnetic susceptibility, and chemical stability, which make them excellent candidates for environmental remediation^9–11^.. However, enhancing the dye-removal performance of mixed ferrites remains challenging, but this can be addressed through surface modification techniques.

Metal ferrites are highly valued for their tunable properties, which make them suitable for diverse applications, including catalysis^12^, biomedical uses^13^, and environmental cleanup^14^. Their magnetic nature allows easy separation from treated water, making them ideal for magnetic-based dye-removal systems^15^.

Multiple factors, including cation distribution, synthesis conditions, particle size, microstructural features, heat treatment, and the chosen preparation method, influence the properties of ferrite materials^16,17^. A wide range of techniques has been employed for ferrite synthesis^17–19^. Among these, the co-precipitation method is considered highly advantageous due to its ability to produce nanoparticles of small size^20^., uniform composition, and high crystallinity, while also ensuring superior yields of ultrafine particles^21^. Within the ferrite family, spinel ferrites represent a critical subgroup. These compounds crystallize in a cubic structure in which magnetic ions occupy both tetrahedral and octahedral sites^22^., imparting distinctive and desirable magnetic properties^23^.

Zinc ferrites, belonging to the class of normal spinel ferrites, exhibit versatile chemical, optical, electrical, and dielectric characteristics. Due to these unique properties, they have attracted significant interest for a wide range of applications, including cancer treatment via magnetic hyperthermia^24^, photocatalytic processes^25^, wastewater treatment^26^, and the development of sensing devices^27^. Recently, ZnFe₂O₄ has been modified with different dopants to enhance its performance in water treatment applications. For instance, Nd³⁺-doped ZnFe₂O₄ ferrites demonstrated up to 98.0% removal of Rhodamine B dye in the presence of hydroperoxide^28^, while Gd³⁺-substituted ZnFe₂O₄ nanoparticles exhibited markedly enhanced catalytic activity^29^. Incorporating lanthanum into ferrite systems has consistently been shown to enhance both catalytic and adsorption performance toward organic dyes. For example, LaFeO₃ perovskites synthesized via the sol–gel method have been effectively applied for dye degradation under low-intensity ultraviolet (UV) irradiation^30^.

Surface modification of metal ferrite with different materials, such as silica^5^, Chitosan^31^, and various polymers to improve their adsorption capacity and overall pollutant removal performance. In particular, Chitosan, a natural biopolymer rich in amino and hydroxyl groups, has been widely used to functionalize ferrite surfaces, thereby enhancing dye adsorption via electrostatic interactions, hydrogen bonding, and chelation^32^.

In the present study, La_0.5_Zn_0.5_Fe₂O₄/LaFeO₃ (LZNF) nanocomposite was synthesized and subsequently coated with Chitosan, and their performance in the removal of the anionic dyes IC and AB 25 was comparatively evaluated. The novelty of this work lies in the design of a La-modified spinel ferrite system, where La₀.₅Zn₀.₅Fe₂O₄ spinel ferrite is the major phase, and LaFeO₃-type perovskite is a minor phase, combined with a Chitosan surface layer to deliberately tune the adsorption interface toward anionic dyes.

## **Reagents and Chemicals**

### **Chemical reagents**

**Fig. 1:**
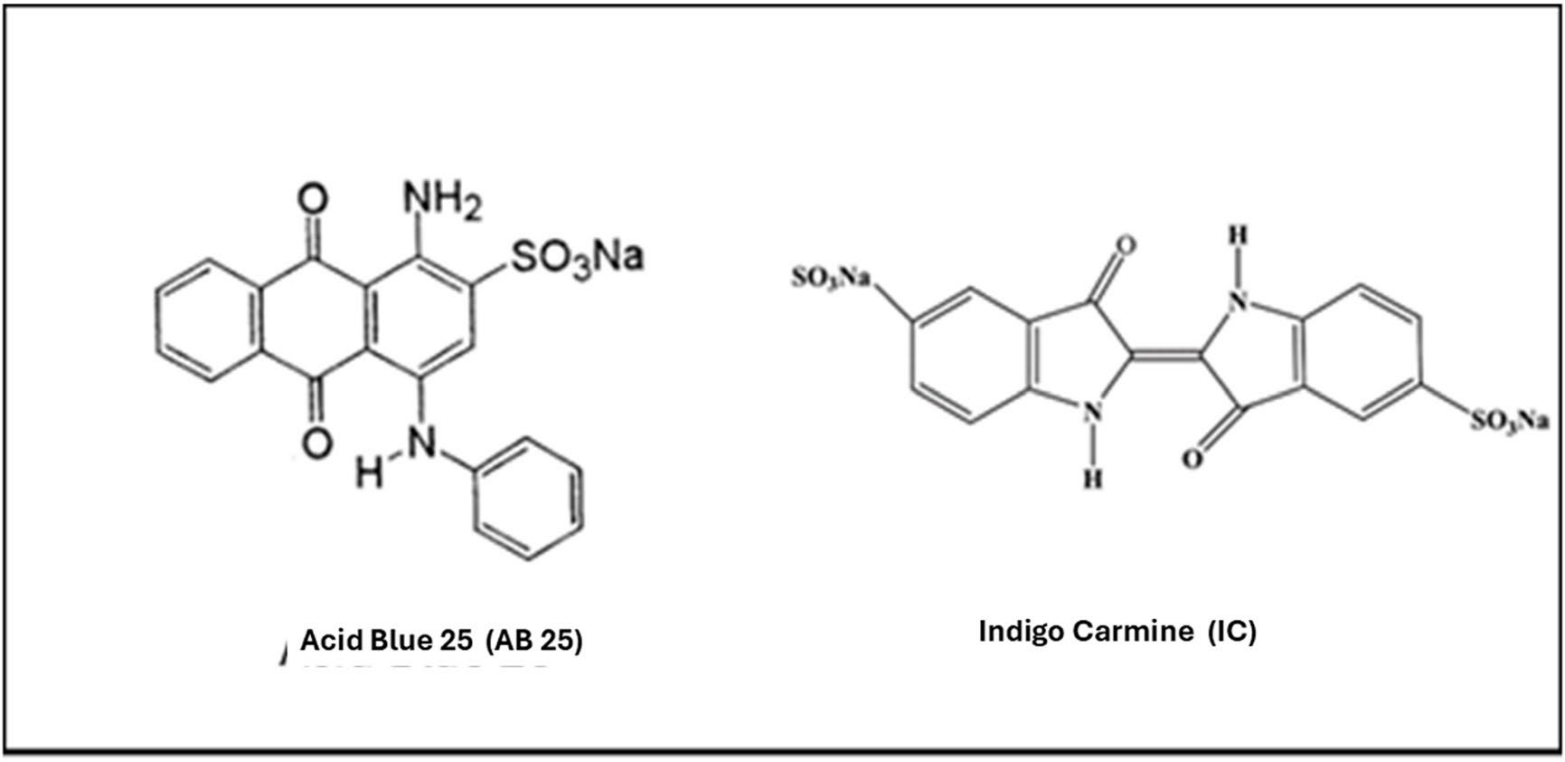
Structure of the Investigated Dyes.

High-purity ferric nitrate nonahydrate (Fe(NO_3_)_3_·9H_2_O, 98%), zinc nitrate hexahydrate (Zn(NO_3_)_2_·6H_2_O, 98%), and lanthanum nitrate hexahydrate (La(NO_3_)_3_·6H_2_O, 97%) were procured from Sigma-Aldrich. Acid blue 25 (C_27_H_35_BrClN_3_, 85%) and indigo carmine (C_16_H_8_N_2_Na_2_O_8_S_2_, 85%) were sourced from Merck. Chitosan (CS) (C_56_H_103_N_9_O_39_) with low molecular weight and a deacetylation degree of 70–90% was also utilized. No additional purification was performed on any of the reagents before use. The molecular structures of the target dye pollutants are presented in Fig. 1.

### **Synthesis of La**_**0.5**_**Zn**_**0.5**_**Fe₂O₄ nanocomposite (LZNF)**

La_0.5_Zn_0.5_Fe₂O₄ nanocomposite was synthesized using the co-precipitation technique^33^. In brief, stoichiometric amounts of Zn(NO₃)₂·6 H₂O (0.5 M), La(NO₃)₃·6 H₂O (0.5 M), and Fe(NO₃)₃·9 H₂O (2 M) were dissolved in distilled water at a 1:2 molar ratio, followed by dropwise addition of NaOH (3 M) until precipitation occurred. The mixture was refluxed at 80 °C for 2 h. Subsequently, the brownish ferrite precipitate was magnetically separated. The collected precipitate underwent multiple washes with deionized water to remove water-soluble impurities, followed by centrifugal separation, and was then dried in an oven at 60 °C for 48 h. Finally, the dried powders were ground using an agate mortar and pestle for several minutes to obtain ultrafine particles.

### Synthesis La_0.5_Zn_0.5_Fe₂O₄/Chitosan nanocomposite (LZNF/CS)

The LZNF/CS nanocomposites were synthesized via an in situ co-precipitation method, using TPP molecules as crosslinking agents for low-molecular-weight Chitosan polymers. Precisely measured quantities of 1.05 mmol La(NO₃)₃·6 H₂O, 1.05 mmol Zn(NO₃)₂·6 H₂O, and 4.2 mmol Fe(NO₃)₃·9 H₂O were transferred into a 100 mL flask, to which 25 mL of double-distilled water was added with vigorous agitation for 60 min. A Chitosan solution was prepared by dissolving 0.375 g of Chitosan powder in 30 mL of 4% glacial acetic acid under stirring for one hour. This Chitosan solution was then added to the metal salt mixture, maintaining continuous stirring. The pH was raised to 11 by dropwise addition of 33% NH₄OH, and stirring was continued for an additional 2 h. Following this, 0.15 g of TPP dissolved in 5 mL of water was introduced, and the mixture was left to cure for 48 h. The resulting brown precipitate was washed repeatedly with distilled water and dried under vacuum at 60 °C for 12 h.

### Characterization techniques

The crystalline structure of the prepared samples was analyzed using powder X-ray diffractometry (GNR APD 2000 PRO diffractometer) with CuK_α_ radiation at 40 kV and 30 mA, scanning a 2θ range of 10° to 70°. Fourier transform infrared spectroscopy (FTIR, JASCO model 4100) with KBr pellets was employed to investigate the molecular structure. Elemental composition analysis was conducted using scanning electron microscopy coupled with energy-dispersive X-ray spectroscopy (SEM-EDX, IT100LA) at a voltage of 20 kV. A vibrating sample magnetometer (VSM, Lake Shore model 7410) was utilized to evaluate the magnetic characteristics of the materials. Surface morphology examination was performed using SEM (Hitachi SU8000 Type II), while transmission electron microscopy (TEM, JEOL JEM-2100) was applied to determine particle dimensions and morphology. The Brunauer-Emmett-Teller (BET) method (Quantachrome Touch WinTM, version 1.21) was used to measure the specific surface area of both LZNF and LZNF/CS samples following a 12-hour degassing process at 120 °C. Pore size distribution profiles, pore volume, and average pore diameter were calculated using the Barrett-Joyner-Halenda (BJH) method.

### Pzc investigation

The point of zero charge (pH_pzc_) was determined to evaluate the influence of pH on the adsorption capacity of the synthesized LZNF and LZNF/CS. Following the methodology described by Rivera et al^34^., the initial pH of 50 mL of 0.01 M NaCl solution was adjusted across a range of 2–12 using 0.1 M HCl or 0.1 M NaOH in separate Erlenmeyer flasks. Subsequently, 0.1 g of the adsorbent material was introduced into each flask. The mixtures were agitated at ambient temperature for 24 h, after which the final pH values were measured. The pHpzc was determined from the intersection of the curves obtained by plotting initial pH against final pH^34,35^.

### Adsorption experiments (Batch methods)

Various adsorption experiments were conducted to evaluate the adsorption performance of the synthesized ferrite materials. For each test, 50-mL Erlenmeyer flasks were prepared containing a predetermined quantity of adsorbent (10–30 mg) and 25 mL of dye solution at a specified concentration. The flasks were placed in a thermostatic water bath shaker and maintained at 120 rpm under controlled temperature conditions. At designated time points, the adsorbent was separated from the solution by combined magnetic attraction and centrifugation. The residual concentrations of AB 25 and IC in the supernatant were quantified by UV–Vis spectrophotometry (Varian Cary 400) at 602 nm and 610 nm, respectively. The dye removal efficiency and the adsorbed dye quantity at time t (q_t_) and at equilibrium (q_e_) were calculated using Eqs. (1–3)^36^.

1 $$\:\mathrm{R}\mathrm{e}\mathrm{m}\mathrm{o}\mathrm{v}\mathrm{a}\text{l }\:\mathrm{e}\mathrm{f}\mathrm{f}\mathrm{i}\mathrm{c}\mathrm{i}\mathrm{e}\mathrm{n}\mathrm{c}\mathrm{y}\:\mathrm{\%}=\frac{{\mathrm{C}}_{\mathrm{o}}-{\mathrm{C}}_{\mathrm{t}}}{{\mathrm{C}}_{\mathrm{o}}}\:\mathrm{x}\:100$$

2 $$\:{q}_{t}=\frac{\left({C}_{o}-{C}_{t}\right)}{m}\:\mathrm{x}\:V\:$$

3 $$\:{q}_{e}=\frac{\left({C}_{o}-{C}_{e}\right)}{m}\:\mathrm{x}\:V\:$$

Where C₀ represents the initial dye concentration, Cₜ denotes the concentration at time t, and Cₑ indicates the equilibrium concentration of the dyes. V (L) represents the volume of the working solution, while m (g) denotes the mass of the nanocomposite adsorbent. The solution pH was adjusted and maintained within the target range (2–12) using a universal buffer system in conjunction with either 0.1 M HCl or 0.1 M NaOH.

## Results and discussion

### Characterization

#### FTIR

Figure 1a depicts the FTIR spectra of LZNF nanocomposite in the 400–4000 cm^-1^ range. The FTIR spectrum of LZNF shows a broad O–H stretching band near 3390 cm⁻¹ and H–O–H bending around 1590 cm⁻¹, both attributed to surface hydroxyls and adsorbed water, along with nitrate residues at 1380 cm⁻¹ and the characteristic spinel lattice vibrations at 558 and 430–455 cm⁻¹ corresponding to tetrahedral and octahedral metal–oxygen bonds, confirming the ferrite structure. In contrast, LZNF/CS exhibit additional features typical of Chitosan, including broad O–H/N–H stretching at 3400 cm⁻¹, C–H stretching near 2990 cm⁻¹, and 1640 cm^− 1^ which are associated with the NH_2_ group bent scissoring. This indicates that CS was successful in coating the LZNF, and that strong C–O–C/C–O vibrations at 1050 cm⁻¹, with a shifted band around 626 cm⁻¹, indicate interactions between the polymer and the ferrite surface. These spectral changes confirm the successful surface modification of La_0.5_Zn_0.5_Fe₂O₄ nanoparticles with Chitosan, while retaining the integrity of the spinel core.

#### XRD

**Fig. 2:**
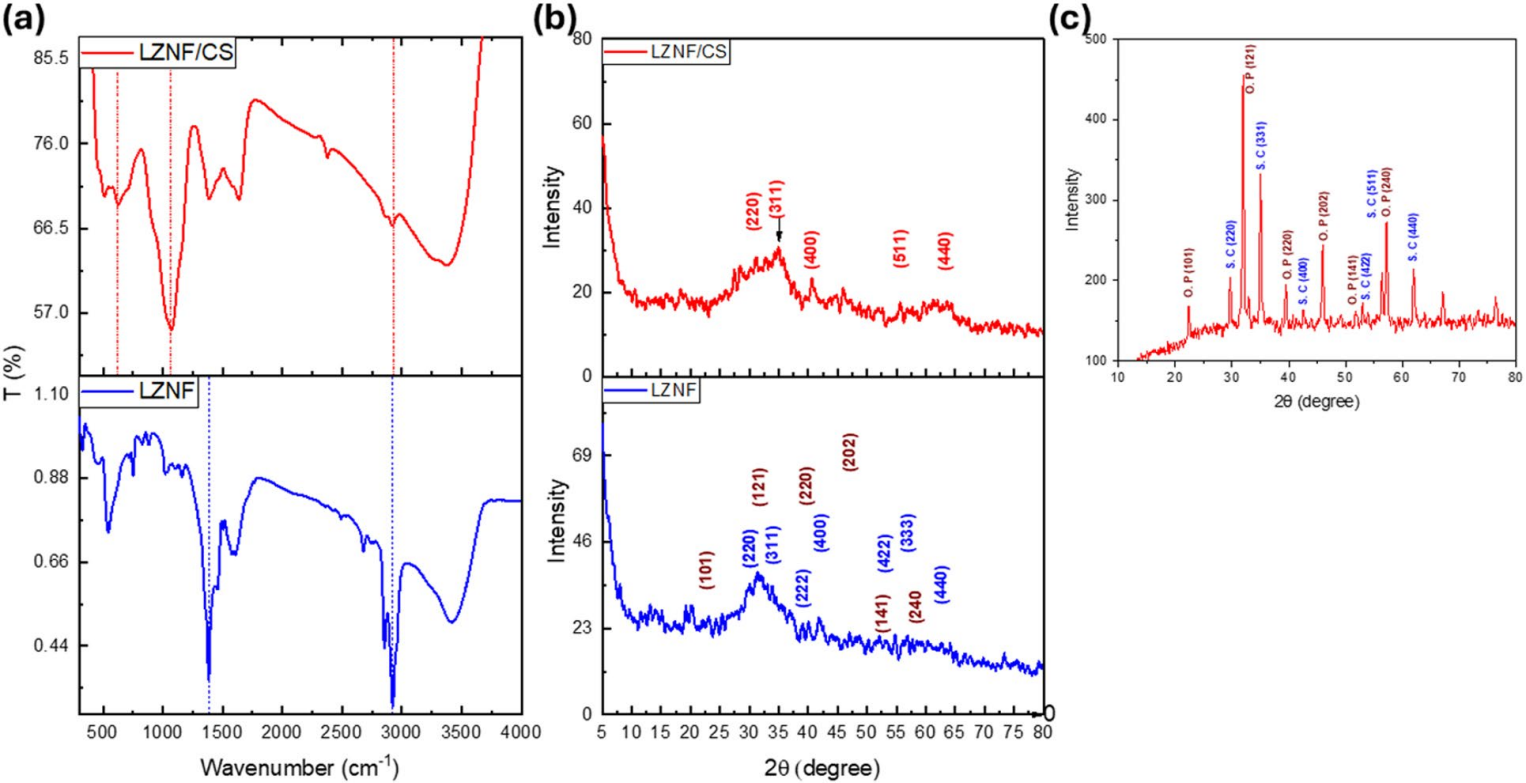
Spectra of a LZNF and LZNF/CS, FTIR (a), XRD (b), and XRD of Annealed LZNF at 900 °C.

X-ray diffraction was employed to investigate the phase composition and crystallinity of the synthesized LZNF and LZNF/CS nanocomposite. As shown in Fig. 2b, the diffraction pattern of LZNF is dominated by a broad spinel-type envelope, with maxima centred at 2θ ≈ 30–33°, 38–41°,  50–52°, and  62–64°. These reflections can be indexed to the (220), (311), (400)/(222), (422), and (440) planes of a cubic spinel ferrite structure, in agreement with JCPDS card no. 73–1720^37^. The pronounced broadening of these peaks indicates nanometric crystallite size and lattice strain.

Superimposed on the spinel pattern, weak additional reflections are observed at 2θ = 22.6°, 32.2°, 39.7°, 46.1°, 52.1°, and 57.5°. These correspond to the (101), (121), (220), (202), (141), and (240) planes of an orthorhombic perovskite phase (LaFeO₃; JCPDS 37–1493)^38^, confirming the presence of a minor secondary phase. This assignment is plausible because the relatively large ionic radius of La³⁺ expands the spinel lattice, producing a slight leftward shift of the spinel reflections.

Upon annealing at 900 °C, the XRD pattern of LZNF (Fig. 2c) exhibits sharper, more intense reflections typical of spinel La_0.5_Zn_0.5_Fe₂O₄ nanoparticles at 2θ = 30.05° (220), 35.40° (311), 43.10° (400), 53.37° (422), 57.02° (511), and 62.51° (440), in agreement with JCPDS card no. 73–1720^37^. In addition, orthorhombic LaFeO₃ remains evident from reflections at 22.6° (101), 32.2° (121), 39.7° (220), 46.1° (202), 52.1° (141), and 57.5° (240), consistent with JCPDS card no. 37–1493^38^. These results clearly confirm that the LZNF consists of two distinct crystalline phases: a cubic spinel ferrite and LaFeO₃ as a secondary phase. Furthermore, TEM/SAED analysis reveals diffuse amorphous rings superimposed on sharp spinel diffraction rings, consistent with the presence of a partially amorphous perovskite-like shell surrounding the spinel nanocrystallites.

After in-situ polymerization of Chitosan on the LFNF nanocomposite, slight peak reduction and broadening were observed, indicating the formation of a Chitosan barrier that may lead to the encapsulation or stabilization of the nanoparticles, resulting in smaller sizes. The average crystallite size was estimated for the (311) peak using the Scherrer equation,

4 $$\:D=\frac{0.9\lambda\:}{\beta\:cos\theta\:}$$

In these expressions, D, λ, β, and θ represent the crystallite size (nm), the wavelength of the X-ray source (0.15406 nm), the full width at half maximum intensity (FWHM) of the diffraction peak, and the Bragg diffraction angle (in radians), respectively. The calculated crystallite size of LZNF was determined to be 10.25 nm. Following the in-situ polymerization of chitosan onto the LZNF nanocomposite, the crystallite dimensions decreased to 8.25 nm. This decrease in particle size can be explained by the development of a chitosan coating layer, which potentially encapsulates or stabilizes the nanoparticles, thereby yielding smaller crystallite dimensions.

####  EDX

**Fig. 3:**
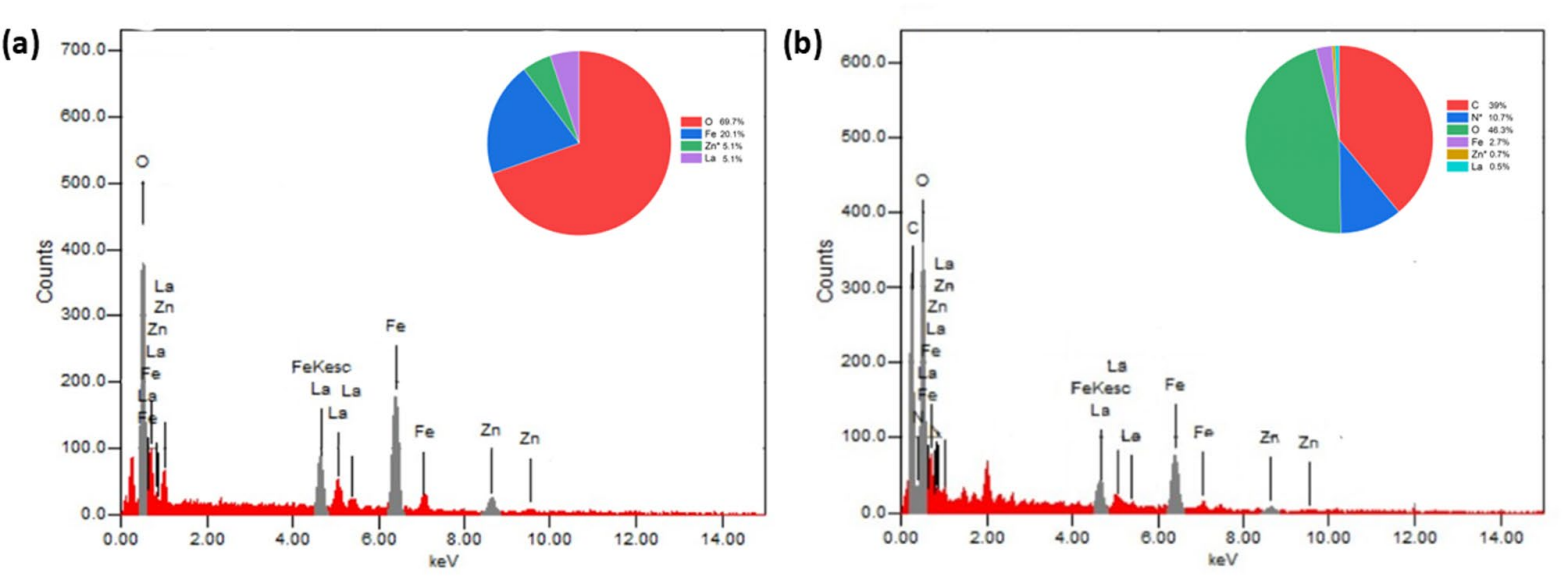
Energy-dispersive X-ray (EDX) spectra of (a) LZNF and (b) LZNF/CS before adsorption.

Energy-dispersive X-ray spectroscopy (EDX) confirmed the elemental composition of the synthesized materials. For LZNF (Fig. 3a), only La, Zn, Fe, and O were detected, consistent with the expected spinel ferrite structure and indicating successful synthesis without detectable impurities. For the LZNF/CS binary nanocomposite (Fig. 3b), the spectrum shows the same metal/oxygen constituents together with clear C and N signals. The presence of N and the enhanced C peak confirm the incorporation of Chitosan into the composite, in agreement with the FTIR findings. The measured atomic percentages of La, Zn, Fe, and O also align with the expected La_0.5_Zn_0.5_Fe_2_O_4_ stoichiometry within the semi-quantitative limits of EDX analysis. 

### VSM

**Fig. 4:**
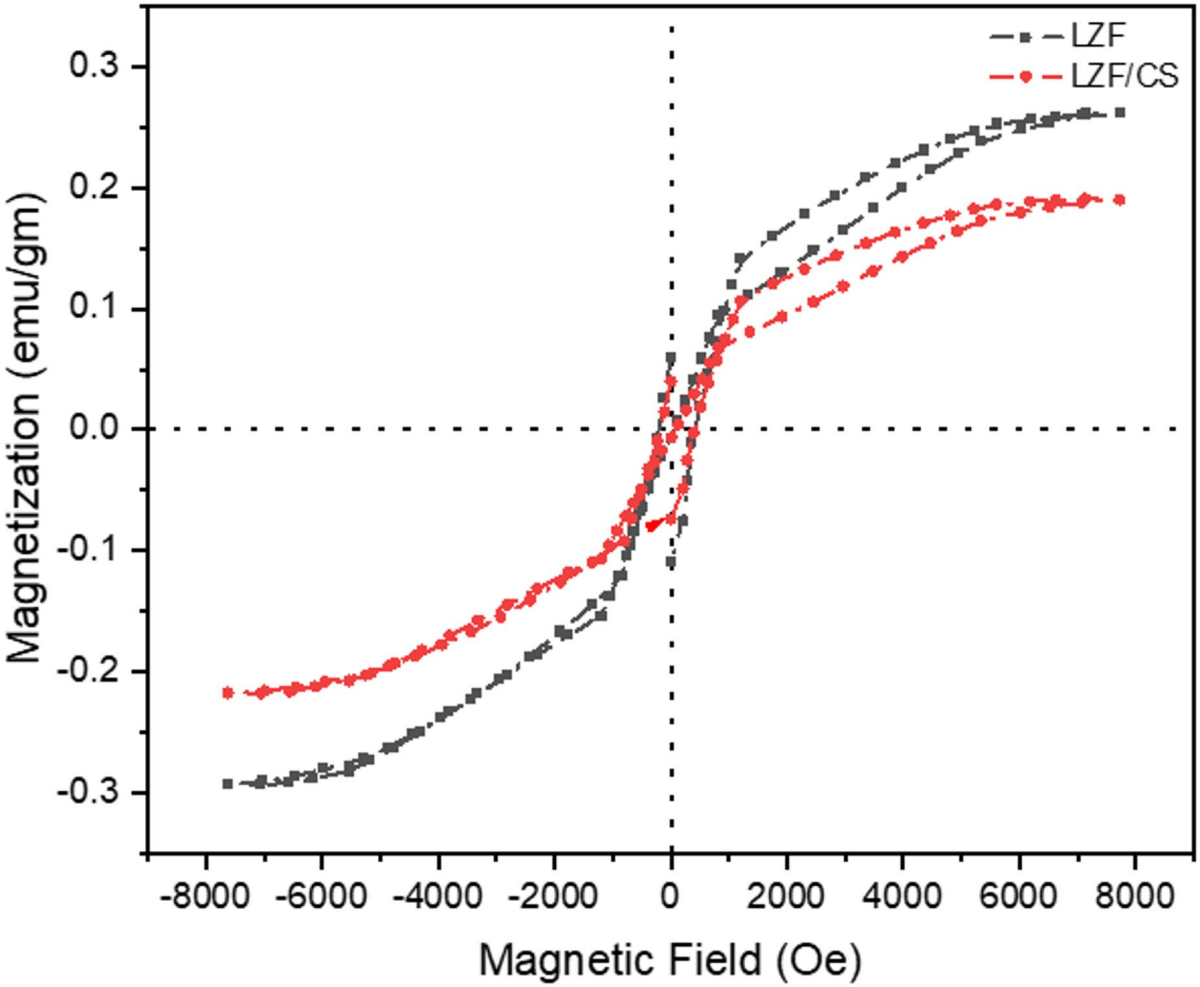
VSM of LZNF (a) and LZNF/CS (b).

The magnetic properties of the synthesized nanoparticles were evaluated using vibrating sample magnetometry (VSM) (Fig. 4), and the results revealed a typical superparamagnetic behaviour for both the uncoated LZNF NPs and the chitosan-coated LZNF/CS, as evidenced by the absence of hysteresis, coercivity, and remanence^39,40^. The magnetisation curves exhibited an S-shaped profile, with low saturation magnetisation values of approximately 0.32 emu/g for LZNF NPs and slightly reduced to 0.22 emu/g after surface modification with Chitosan. This reduction in magnetic response can be attributed to the non-magnetic polymeric shell, which decreases the overall magnetic content per unit mass and induces spin disorder at the nanoparticle interface.

#### Surface morphology

**Fig. 5:**
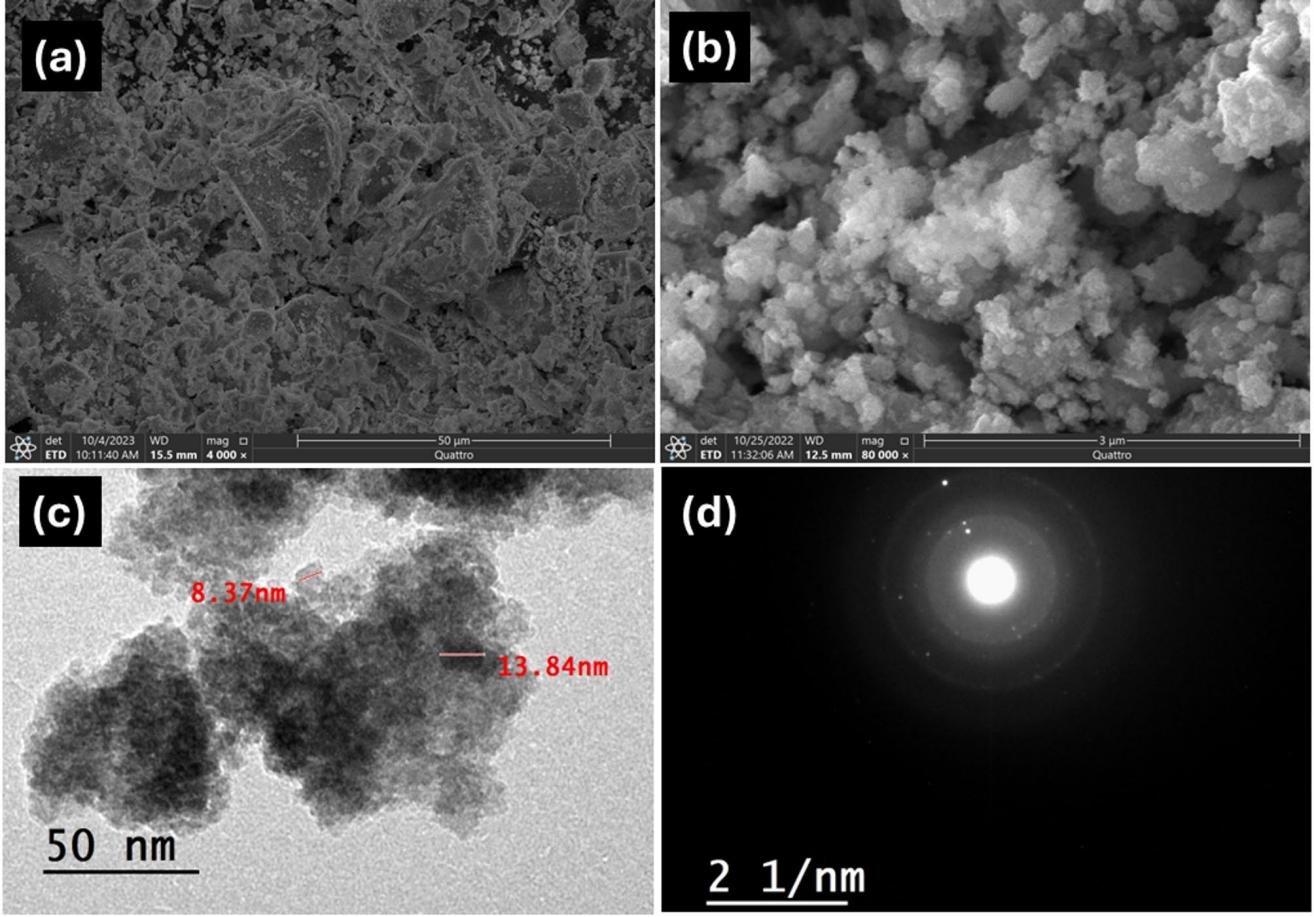
SEM of LZNF (a, b), TEM of LZNF(c), and SAED of LZNF (d).

The SEM micrograph Fig. 5(a, b) shows a highly agglomerated powder of irregular, platelet-like particles with rough surfaces decorated by much finer particulates, giving a hierarchical texture. Agglomeration arises primarily from the high surface energy of the nanosized particles and capillary forces during drying; moreover, transient dipole–dipole interactions between superparamagnetic moments can still promote clustering at close contact. The TEM image in Fig. 5c reveals ramified, quasi-spherical agglomerates composed of rounded to slightly irregular nanocrystallites. Individual primary domains are predominantly 8–14 nm in size, in good agreement with the crystallite size inferred from XRD. The SAED pattern Fig. 5d shows a bright transmitted beam surrounded by concentric, slightly diffuse rings with occasional spotty intensities, characteristic of a nanocrystalline polycrystalline material with a thin amorphous surface layer contributing to the background.

#### BET

**Fig. 6:**
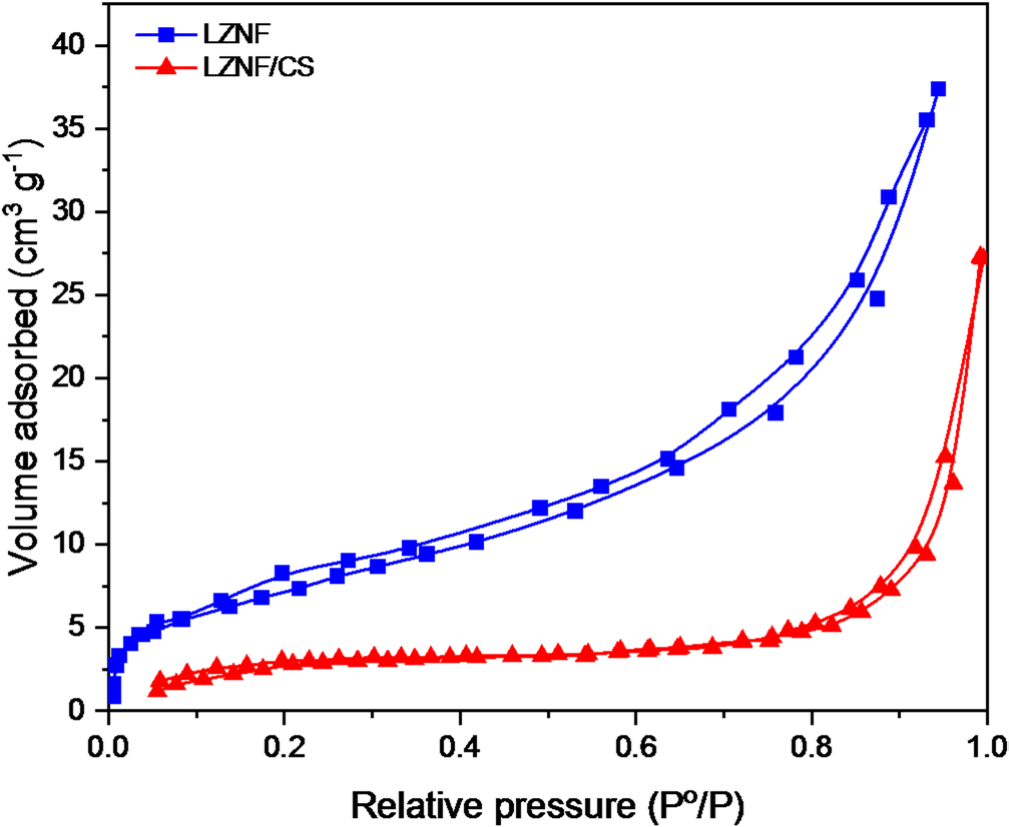
N_2_ adsorption-desorption isotherm curves of LZNF and LZNF/CS at 77 K.

The specific surface areas of LZNF and LZNF/CS and their pore-size characteristics were obtained from N₂ adsorption–desorption (BET). The isotherms in Fig. 6 are type IV with a hysteresis loop at P/P₀ > 0.4, confirming a mesoporous structure^41^. LZNF shows a BET area of 72.21 m²/g, larger than that of the LZNF/CS nanocomposite (31.65 m²/g), indicating that Chitosan coating reduces the accessible surface, likely by partially blocking ferrite pores. Consistently, the total pore volume and average pore diameter decrease from 0.197 to 0.124 cm³/g and from 5.33 to 3.10 nm, respectively. These pore diameters place both materials in the mesoporous region, which is favourable for adsorption.

### Adsorption study for IC and AB 25

The main goal of this research is to develop effective adsorbents for the removal of toxic dyes that pose risks to human health and contaminate water. The adsorption efficiency of LZNF and the LZNF/CS nanocomposite as novel adsorbents for wastewater treatment was evaluated using Indigo Carmine (IC) and Acid Blue 25 (AB 25) as model anionic dyes. In contrast, other cationic dyes, such as Methylene Blue, Methyl Green, and Acridine Orange, showed no significant adsorption onto these materials under different conditions.

#### Effect of pH

**Fig. 7:**
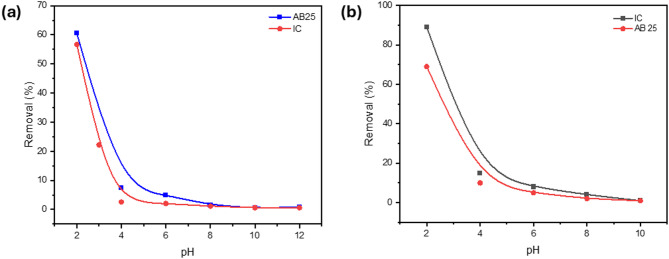
Adsorption efficiency of (a) LZNF and (b) LZNF/CS for IC (45.8 and 41.9 mg L⁻¹) and AB25 (57.0 and 49.9 mg L⁻¹), respectively, at different pH levels of the dye solutions, using 0.024 g of LZNF and 0.030 g of LZNF/CS.

The pH level plays a crucial role in regulating adsorption. Both the adsorbate dyes (Indigo Carmine, IC, and Acid Blue 25, AB 25) and the adsorbents (LZNF and LZNF/CS nanocomposite) contain various surface functional groups that can gain or lose protons (H⁺) depending on the medium pH. To optimize pollutant removal, the adsorption of IC and AB 25 was investigated over a wide pH range (2–12), as shown in Fig. 7.

The results revealed that the adsorption of both dyes onto LZNF and LZNF/CS nanocomposite decreased progressively with increasing pH. Maximum dye removal occurred at acidic conditions (pH = 2), while adsorption efficiency dropped significantly at higher pH values, reaching a minimum in alkaline conditions. This behaviour can be explained by the point of zero charge (PZC) of each adsorbent, which was measured to be 8.1 for LZNF and 5.9 for LZNF/CS. At pH values below the PZC (i.e., < 8.1 for LZNF and < 5.9 for LZNF/CS), the surface of the adsorbents becomes positively charged, favouring electrostatic attraction with the negatively charged anionic dyes (IC and AB 25). Conversely, at pH values above the PZC, the adsorbent surface carries a negative charge, leading to electrostatic repulsion and reduced adsorption efficiency. This also explains why both adsorbents show negligible adsorption of cationic dyes, since electrostatic attraction is absent under the studied conditions.

Furthermore, a difference was observed between the two adsorbents: LZNF maintained significant adsorption in aqueous medium, while LZNF/CS exhibited much lower efficiency under similar conditions. This discrepancy is attributed to LZNF’s higher PZC value (8.1) compared to LZNF/CS (5.9). Specifically, the removal efficiency of IC reached 89% at pH 2 on LZNF but declined sharply at higher pH. Similarly, AB 25 removal on the LZNF/CS composite was 69% at pH 2, with efficiency decreasing markedly as the solution became less acidic.

The highest adsorption capacities for IC and AB 25 on both LZNF and LZNF/CS were achieved at **pH 2**. At this level, the adsorbent surfaces are strongly positively charged, maximizing the electrostatic attraction between the anionic sulfonate groups of the dyes and the positive metal surface sites. Conversely, at higher pH values, removal efficiency decreases due to reduced surface protonation and weakened electrostatic interactions.

While such highly acidic conditions may seem less practical for general wastewater applications, many industrial dyeing and finishing effluents are already acidic, and pre-acidification is a standard practice before coagulation or adsorption steps. Therefore, operation at moderately acidic levels (pH 3–7) remains feasible, though it necessitates a trade-off between peak treatment efficiency and the chemical costs associated with pH adjustment.

#### Influence of adsorbent dosage

**Fig. 8:**
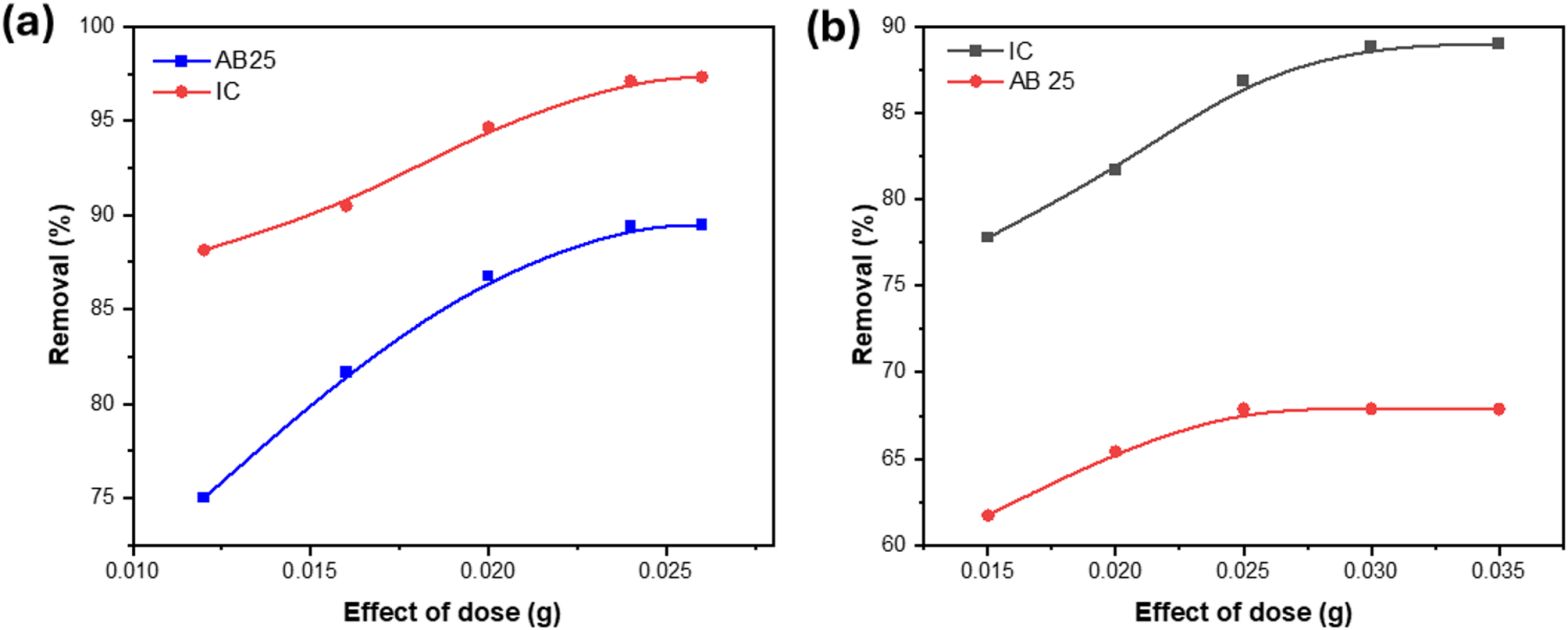
Effect of adsorbent dose on the removal efficiency of LZNF for IC (45.8 mg L⁻¹) and AB25 (57.0 mg L⁻¹) in aqueous solution (a), and of LZNF/CS for IC (41.9 mg L⁻¹) and AB25 (49.9 mg L⁻¹) at pH = 2 (b).

The influence of adsorbent dosage on the removal of IC and AB 25 dyes was examined using LZNF and LZNF/CS nanocomposite at 30 °C, with doses ranging from 0.01 to 0.03 g, as presented in Fig. 8. For LZNF in aqueous medium, increasing the dosage from 0.01 to 0.026 g resulted in enhanced removal percentages for IC and AB 25, rising from 88.2% to 98.0% and from 75.5% to 87.5%, respectively. Similarly, when the LZNF/CS nanocomposite dosage was increased from 0.01 to 0.035 g under acidic conditions (pH = 2), the removal percentages for IC and AB 25 improved from 77.7% to 88.9% and from 61.7% to 67.8%, respectively. This enhancement can be attributed to the increased number of available active sites for both dyes as the adsorbent quantity increased. Beyond an adsorbent dose of 0.035 g, the removal efficiency remained relatively constant for both dyes, suggesting that no additional adsorption took place. This plateau indicates that the system had achieved adsorption equilibrium.

#### Effect of dye concentration

**Fig. 9:**
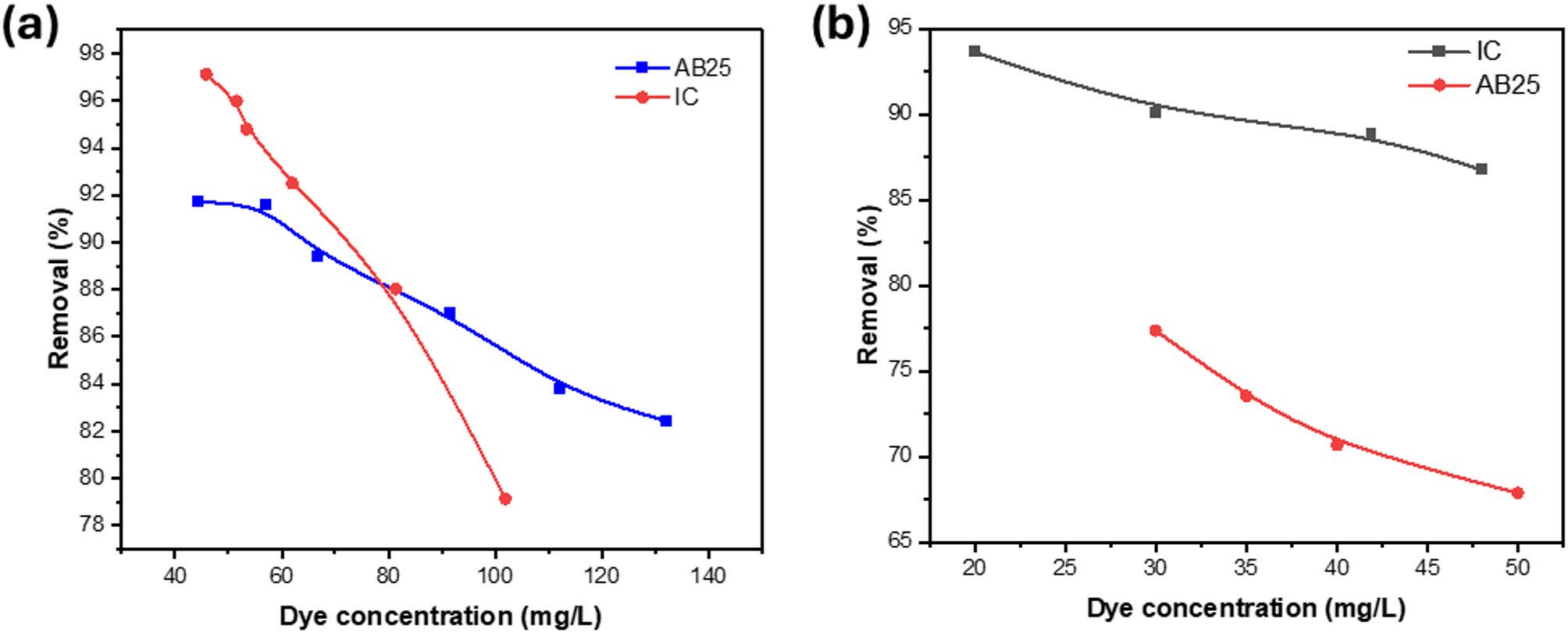
Adsorption efficiency of LZNF (0.024 g) (a) and LZNF/CS(0.03 g) (b) for varying concentrations of IC and AB 25 in aqueous medium and pH 2, respectively, at 30 °C.

To examine the effect of initial dye concentration on adsorption, IC concentrations were varied from 40 to 110 mg/L for LZNF and 20–50 mg/L for LZNF/CS, while AB 25 concentrations were adjusted from 45 to 150 mg/L for LZNF and 30–49.9 mg/L for LZNF/CS. Using LZNF (0.024 g), the removal efficiency decreased from 98% to 75.5% for IC and from 92.5% to 83.2% for AB 25 (Fig. 9a). In contrast, applying LZNF/CS (0.03 g) at pH 2 caused a drop in removal efficiency from 93.6% to 86.7% for IC and from 77.3% to 67.8% for AB 25 (Fig. 9b). At low dye concentrations, both adsorbents offer abundant accessible active sites, yielding higher removal efficiencies. As the dye concentration increases, these active sites become progressively saturated, reducing the adsorption capacity and consequently lowering the removal efficiencies for both IC and AB 25.

#### Thermodynamic study

**Fig. 10:**
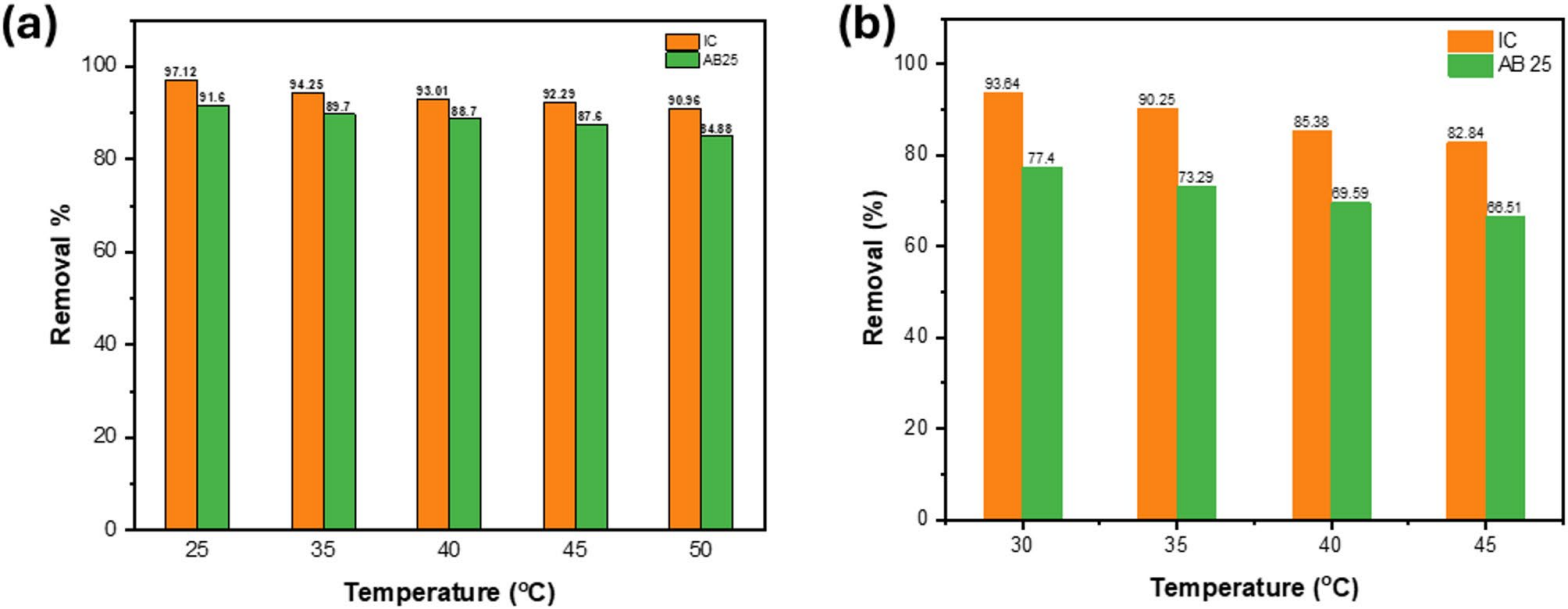
Effects of temperature on the removal of IC (45.8, 30 mg/L) and AB25 (57, 49 mg/L) using LZNF(aqueous medium, 0.024 g) and LZNF/CS (pH = 2,0,03 g).

Reaction temperature is a key factor influencing the adsorption rate. The effect of temperature on dye removal was investigated for LZNF and LZNF/CS, with LZNF adsorption in aqueous medium and LZNF/CS under acidic conditions (pH 2). Initial concentrations of IC (45.8, 47.0 mg/L) and AB 25 (57, 49.5 mg/L) were used, with adsorbent doses of 0.024 g and 0.03 g, respectively. As shown in Fig. 10 (a, b), the removal efficiency of IC and AB 25 decreased from 97.2% to 90.3% and from 91.6% to 84.8% for LZNF, and from 93.5% to 82.8% and from 77.4% to 66.5% for LZNF/CS, as the temperature increased from 25 to 50 °C. This decline confirms the exothermic nature of the adsorption process, as higher temperatures weaken surface interactions and increase the mobility of the dye molecules, thereby reducing adsorption.

Thermodynamic parameters for IC and AB 25 adsorption were calculated at different temperatures using Eqs. (5, 7) ^5^,

5 $$\:\mathrm{ln}{k}_{d}=\frac{\varDelta\:{S}^{o}}{R}-\frac{\varDelta\:{SH}^{o}}{RT}\:$$

6 $$\:{k}_{d}=\frac{{q}_{e}}{{C}_{e}}\:$$

7 $$\:\varDelta\:{G}^{o}=\varDelta\:{H}^{o}-T\varDelta\:{S}^{o}$$

Where *k*_*d*_ is the distribution constant (L/g), *R* is the gas con7stant (8.314 J·mol⁻¹·K⁻¹), and *T* is the absolute temperature (K). The calculated values are listed in Table 2. Negative ΔG° values indicate that adsorption of both dyes onto LZNF and LZNF/CS is spontaneous, while negative ΔH° values confirm the exothermic nature of the process. Moreover, the negative ΔS° values indicate a reduction in randomness at the solid–liquid interface, as dye molecules become more ordered on the adsorbent surface Tables 4 and 5.

**Table 1:** Thermodynamic parameters for IC and AB 25 adsorption on LZNF and LZNF/CS at various temperatures.

Adsorbent	T (K)	$\:\varDelta\:\boldsymbol{G}^\circ\:\:$(KJ/mol)		$\:\varDelta\:\boldsymbol{H}^\circ\:$(KJ/mol)		$\:\varDelta\:\boldsymbol{S}^\circ\:$(KJ/mol)	
		**AB25**	**IC**	**AB25**	**IC**	**AB25**	**IC**
LZNF	298	−5.49	−8.26	−20.92	−38.16	−51.53	−101.10
	308	−5.07	−6.69				
	313	−4.89	−6.26				
	318	−4.59	−6.08				
	323	−4.14	−5.71				
LZNF/CS	308	−2.96	−6.06	−28.48	−59.79	−84.22	−177.31
	313	−2.54	−5.18				
	318	−2.12	−4.29				
	323	−1.70	−3.40				

#### Adsorption kinetics

**Fig. 11:**
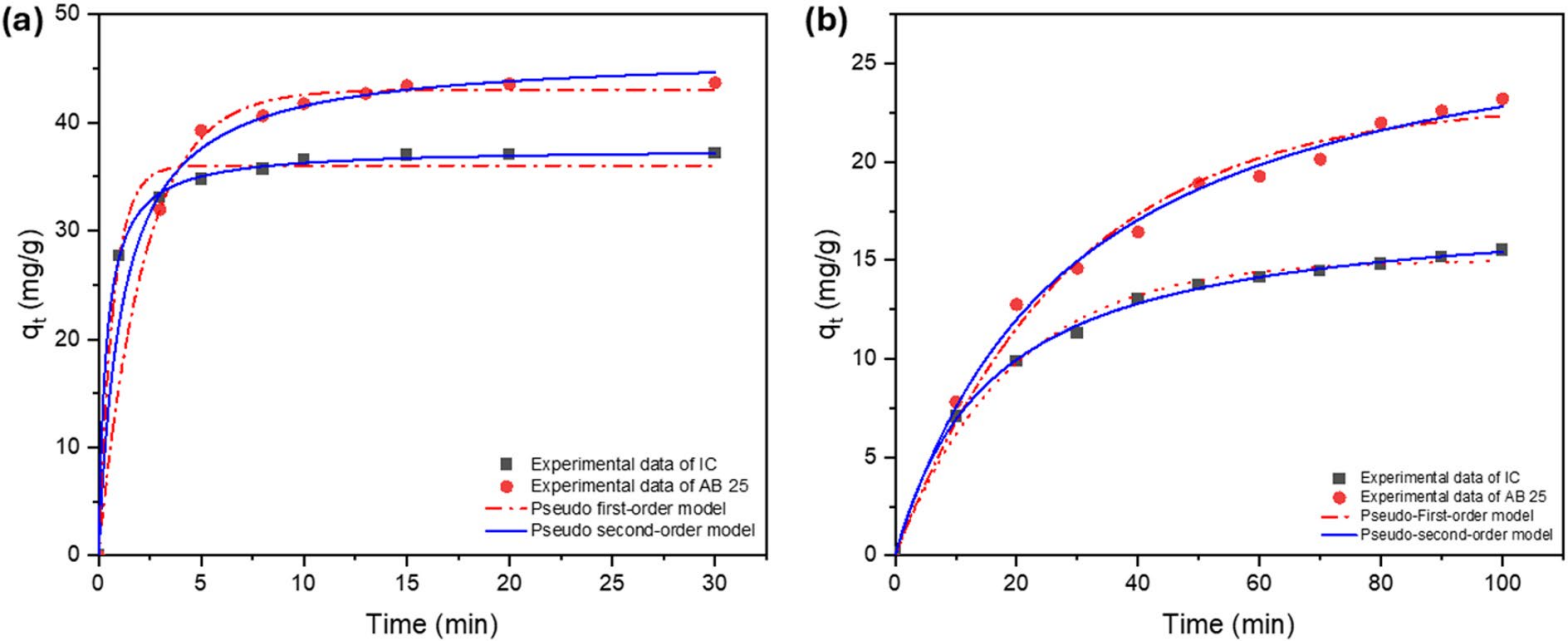
Non-linear plots of pseudo-first and second-order kinetics for IC and AB 25 adsorption on LZNF(a) and on LZNF/CS nanocomposite (b).

The adsorption kinetics of IC and AB 25 onto LZNF and LZNF/CS nanocomposites were analyzed using three kinetic models: pseudo-first-order, pseudo-second-order, and intraparticle diffusion. The non-linear expressions of the first and second models are represented by Eqs. (8) and (9), respectively^42–44^,. In these equations, k₁ (min⁻¹) and k₂ (g/mg min) denote the rate constants for pseudo-first and pseudo-second-order kinetics, while qₑ and qₜ represent the adsorbed amounts of each dye (IC and AB 25) at equilibrium and at time t (min), respectively. The model with the best fit to the experimental data is identified by the highest R² value. Figure 11a and b illustrate the nonlinear pseudo-first-order and pseudo-second-order kinetic curves describing the adsorption of IC and AB 25 onto LZNF and LZNF/CS nanocomposites, respectively, and the corresponding parameter values are summarized in Table 2. The pseudo-second-order model yields the highest determination coefficients for all systems, indicating superior agreement with the experimental data for both IC and AB 25 adsorption on LZNF and LZNF/CS nanocomposites. In addition to the higher $\:{R}^{2}$ values, the calculated equilibrium capacities (q_e, calc_) obtained from the pseudo-second-order fits are very close to the experimental qe values, further confirming that this model provides the most reliable description of the adsorption kinetics under the studied conditions.

**Fig. 12:**
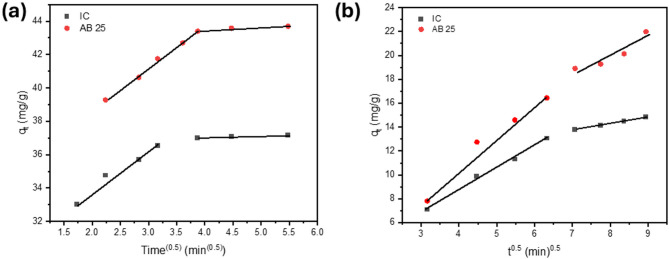
Intra-particle diffusion kinetics model for IC and AB 25 adsorption on LZNF(a) and on LZNF/CS nanocomposite (b).

. During adsorption, dyes are typically transported from the bulk solution to the adsorbent surface through two primary mechanisms: external mass transfer (boundary-layer diffusion) and intraparticle diffusion (pore diffusion). The intraparticle diffusion model helps identify which transport mechanism predominantly controls the adsorption rate. Therefore, the kinetic behavior of IC and AB 25 adsorption was further examined using the intraparticle diffusion equation (Eq. (10))^4,44–46^. The intraparticle diffusion rate constant (kₚ, mg/g min⁰·⁵) and the boundary layer thickness constant (C, mg/g) were obtained from the slope and intercept of the qₜ versus t⁰·⁵ plot, as shown in Fig. 12 and summarized in Table 2. According to this model, a plot passing through the origin suggests that intraparticle diffusion is the primary rate-controlling mechanism for dye adsorption. However, the plot did not pass through the origin (Fig. 12), suggesting that intraparticle diffusion was not the only rate-limiting step. This indicates that additional mechanisms beyond intraparticle diffusion also influence the overall adsorption rate. The adsorption of IC and AB 25 onto both LZNF and LZNF/CS nanocomposites proceeds through two stages: the initial stage represents boundary-layer diffusion (external mass transfer), whereas the subsequent stage corresponds to inner-surface diffusion (intraparticle diffusion). Table 2 demonstrates that external diffusion on the adsorbent surface proceeds rapidly, followed by slower diffusion into the internal surface.

8 $$\:{q}_{t}={q}_{e}\left(1-{e}^{-{k}_{1}t}\right)\:$$

9 $$\:{q}_{t}=\frac{{k}_{2}{q}_{2}^{2}t}{1+{q}_{e}{k}_{2}t}\:$$

10 $$\:{q}_{t}={k}_{p}{t}^{0.5}+C\:$$

**Table 2:** Kinetic parameters for IC (45.8, 30 mg/L) and AB25 (57, 49 mg/L) adsorption on LZNF (0.024 g) and LZNF/CS nanocomposite (0.03 g) at aqueous medium and at pH 2, respectively.

Kinetics parameter	parameters	LZNF		LZNF/CS	
		IC	AB 25	IC	AB 25
**Pseudo-first order**	q_e expl_ (mg/g)	37.16	43.91	16.55	25.00
	q_e cal_ (mg/g)	14.985	27.608	15.02	23.05
	K_1_ (min^− 1^)	0.31836	0.2839	0.052	0.034
	R^2^	0.96788	0.9877	0.990	0.986
**Pseudo-Second order**	q_e expl_ (mg/g)	37.16	43.91	16.55	25.00
	q_e cal_ (mg/g)	37.70	45.31	17.88	29.46
	K_1_ (min^− 1^)	0.06936	0.0246	0.0035	0.0011
	R^2^	0.999	0.999	0.998	0.994
**Intraparticle diffusion**	K_p1_ (mg/g min^0.5^)	3.861	5.214	1.50	2.192
	C (mg/g)	25.085	25.121	1.36	0.189
	R^2^	0.900	0.820	0.991	0.991
	K_p2_ (mg/g min^0.5^)	0.095	0.176	0.461	1.307
	C (mg/g)	36.635	42.744	9.757	7.342
	R^2^	0.979	0.933	0.999	0.879

#### Adsorption isotherms

Adsorption isotherms show how the amount of adsorbate on a surface changes with its concentration. By collecting experimental data at constant temperature and pressure, specific isotherm models can be used to estimate parameters such as adsorption capacity and affinity constants. In this study, Langmuir, Freundlich, Temkin, and Dubinin-Radushkevich (D-R) isotherm models were used to analyze the adsorption data of IC and AB 25 dyes on LZNF and LZNF/CS nanocomposites. The experimental data were examined using the Langmuir isotherm model (Eq. (11)), the Freundlich isotherm model (Eq. (12)), the Temkin model (Eq. (13)), and the Dubinin-Radushkevich (D-R) model (Eqs. (14–16)).

11 $$\:\frac{{C}_{e}}{{q}_{e}}=\frac{1}{\:{K}_{L}{q}_{max}}+\frac{{C}_{e}}{{q}_{max}}$$

12 $$\:ln{q}_{e}=\frac{1}{n}Ln{C}_{e}+Ln{K}_{f}$$

13 $$\:{q}_{e}=\frac{\mathrm{R}\mathrm{T}}{b}\:Ln{K}_{t}+\frac{\mathrm{R}\mathrm{T}}{b}\:Ln{C}_{e}$$

14 $$\:{lnq}_{e}=Ln{q}_{m}-\beta\:{\epsilon\:}^{2}\:\:$$

15 $$\:\epsilon\:=RTln\left(\frac{1}{{C}_{e}}+1\right)$$

16 $$\:E=\frac{1}{\sqrt{2\beta\:}}$$

The parameter q_max_ defines the maximum monolayer adsorption capacity in mg per gram, and k_L_ is the Langmuir isotherm constant measured in L per mg. The value n reflects the adsorption intensity, while k_F_ is the Freundlich constant with units mg per gram multiplied by (L per mg) raised to the power of 1/n. K_t_ represents the equilibrium binding constant (L/g) associated with the highest binding energy. The variable b indicates the heat of adsorption in kJ per mole, R is the universal gas constant (8.314 J/mol·K), and T is the absolute temperature in Kelvin. The Polanyi potential, ε (J/mol), is used in the model, whereas qs (mg/g) is the theoretical saturation capacity of each dye on the adsorbent. Finally, the constant β, with units mol² kJ², is connected to the adsorption energy E.

**Fig. 13:**
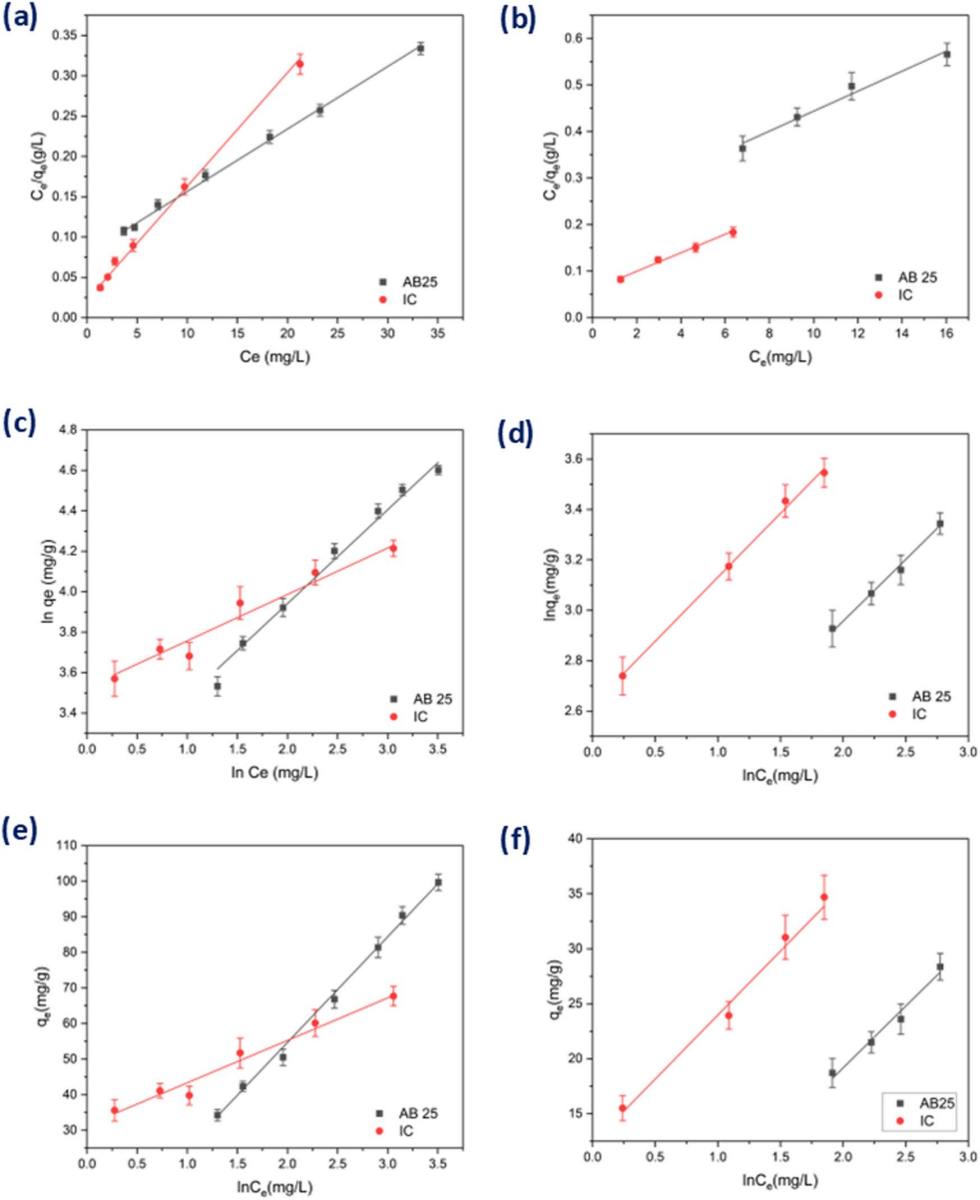
linear Langmuir, Freundlich, isotherm, and Temkin isotherm plots for IC and AB 25 adsorption on LZNF (a, c, d) and on LZNF/CS nanocomposite (b, d,f), respectively.

According to the Langmuir model, the adsorbent surface is assumed to be uniform, with all adsorption sites being identical. Adsorption occurs predominantly as a monolayer of adsorbate molecules, with no interaction between adsorbed molecules. The adsorption data of both dyes on LZNF exhibited an excellent fit with the Langmuir model (Fig. 13a, b), as indicated by the high correlation coefficients (R² values). The maximum adsorption capacities (q_max_) obtained from the model were 71.22 mg g⁻¹ for IC and 125.5 mg g⁻¹ for AB25(Table 3).

In contrast, the Freundlich model accounts for multilayer adsorption on heterogeneous surfaces and allows for reversible adsorption. The adsorption behaviour of both dyes on the LZNF/Cs nanocomposite was better described by the Freundlich model(Fig. 13c, d), suggesting that the Chitosan coating introduced surface heterogeneity and facilitated multilayer adsorption. Moreover, the Freundlich constants (n) for IC and AB25 were greater than one, confirming that the adsorption process was favourable.

The adsorption data for IC and AB 25 on LZNF and LZNF/CS were analyzed using the Temkin isotherm model (Fig. 13(e, f), which assumes that the heat of adsorption decreases linearly with surface coverage due to adsorbate–adsorbent interactions. That adsorption occurs with a uniform distribution of binding energies up to a maximum value. The adsorption energy parameter *b* calculated for IC and AB 25 on both adsorbents (Table 3) suggests that the process is primarily governed by weaker van der Waals or electrostatic interactions, indicating physisorption. The Dubinin–Radushkevich (D–R) model further supports this interpretation, which classifies adsorption type based on the mean free energy (*E*). Specifically, when *E* < 8 kJ mol⁻¹, physisorption dominates; values between 8 and 16 kJ mol⁻¹ correspond to ion-exchange mechanisms, while *E* > 16 kJ mol⁻¹ indicates chemisorption^47^. The low *E* values obtained in this study (Table 3) clearly confirm that the adsorption of both dyes is primarily physical.

**Table 3:** Adsorption isotherm parameters for IC (45.8, 30 mg/L) and AB25 (57, 49 mg/L) adsorption on LZNF (0.024 g) and LZNF/CS nanocomposite (0.03 g) at aqueous medium and at pH 2, respectively.

Isotherm model	parameters	LZNF		LZNF/CS	
		IC	AB 25	IC	AB 25
**Langmuir**	**q**_**max**_ (mg/g)	71.22	125.5	50.23	45.41
	**K**_**L**_ (L/mg)	0.633	0.097	0.322	0.095
	**R** ^**2**^	0.992	0.991	0.981	0.982
**Freundlich**	**n**	4.32	2.179	2.01	2.03
	**K**_**F** (_mg g^− 1^)(L mg^− 1^)^1/n^	33.78	20.28	13.73	7.17
	**R** ^**2**^	0.978	0.982	0.996	0.993
**Temkin**	**k**_**t**_ **(**L/mg)	13.90	0.852	2.871	0.745
	**b** (kJ/mol)	0.207	0.084	0.212	0.221
	**R** ^**2**^	0.979	0.996	0.981	0.981
**Dubinin-Radushkevich**	**q**_**s**_ (mg/g)	53.39	83.93	33.11	29.07
	**β** (mol²/J²)	2.96 × 10^− 7^	2.80 × 10^− 6^	3.3 × 10^− 7^	3.37 × 10^− 6^
	**E** (kJ/mol)	1.30	0.448	1.16	0.354
	**R** ^**2**^	0.757	0.889	0.95	0.910

#### Adsorbent reusability

**Fig. 14:**
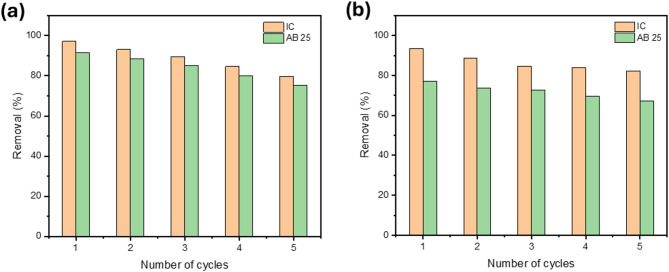
Effect of recycling for IC (45.8, 30 mg/L) and AB25 (57, 49 mg/L) adsorption on LZNF (0.024 g) (a) and LZNF/CS nanocomposite (0.03 g) at aqueous medium and at pH 2 (b).

The reusability of dye adsorbents is essential for their sustainable application in wastewater treatment, ensuring both cost-effectiveness and economic feasibility. To evaluate the reusability of the LZNF and LZNF/CS nanocomposites, the spent adsorbents were recovered from solution by centrifugation and regenerated by shaking in 0.1 M NaOH. This was followed by centrifugation, washing, and drying at 40 °C, a process that further supports the predominance of physical adsorption of these dyes. The regenerated nanocomposites were then subjected to consecutive adsorption–desorption cycles for each dye individually. Over five successive cycles (Fig. 14), adsorption efficiency declined only slightly. For LZNF, removal efficiency decreased from 97.12% to 79.81% for indigo carmine and from 91.6% to 75.36% for acid blue. Similarly, for LZNF/CS, efficiency decreased from 93.5% to 82.2% for indigo carmine and from 77.2% to 65.6% for acid blue. Although a gradual reduction in performance was observed, the results confirm that both nanocomposites retain considerable adsorption capacity across multiple cycles, highlighting their potential as effective and reusable adsorbents for wastewater treatment.

#### Comparison with other adsorbents

The adsorption performance of LZNF and LZNF/CS nanocomposites was assessed by comparing their maximum adsorption capacities for IC and AB 25 dyes with those of previously reported adsorbents. As shown in Table 5, LZNF, followed by LZNF/CS, demonstrated notably high adsorption capacities. These findings highlight the strong potential of these novel nanocomposites as effective adsorbents for dye removal from wastewater.

**Table 4:** Comparison of Adsorption Capacities of Adsorbents for IC and AB 25 Dye Removal from Simulated Wastewater.

Dyes	Materials	Maximum adsorption capacity q_m_(mg/g)	References
**Acid blue 25**	Peat	14.4	^48^
	Modified silica	45.8	^49^
	Raw diatomite	21.41	^50^
	Chitosan-supported cobalt ferrite nanocomposite	63.34	^51^
	NiFe2O4-graphene oxide	104.16	^52^
	Chitosan–TiO_2_/cellulose acetate	76.22	^53^
	Zeolite–CTAB	112.4	^54^
	Nano magnetic manganese ferrite	156	^55^
	LZNF	125.5	This work
	LZNF/CS	45.41	This work
**Indigo carmine**	Rice husk ash	29.3	^56^
	MgFe_2_O_4_	46.08	^57^
	CuFe_2_O_4_	52.06	^58^
	Chitosan-graphene oxide	29.7	^59^
	Activated carbon-based KOH	13.405	^60^
	Activate carbon	31.02	^61^
	Carbon nanotubes	93	^62^
	LZNF	71.22	This work
	LZNF/CS	50.23	This work

#### Characterization of LZNZ and LZNF/CS after Adsorption

**Fig. 15:**
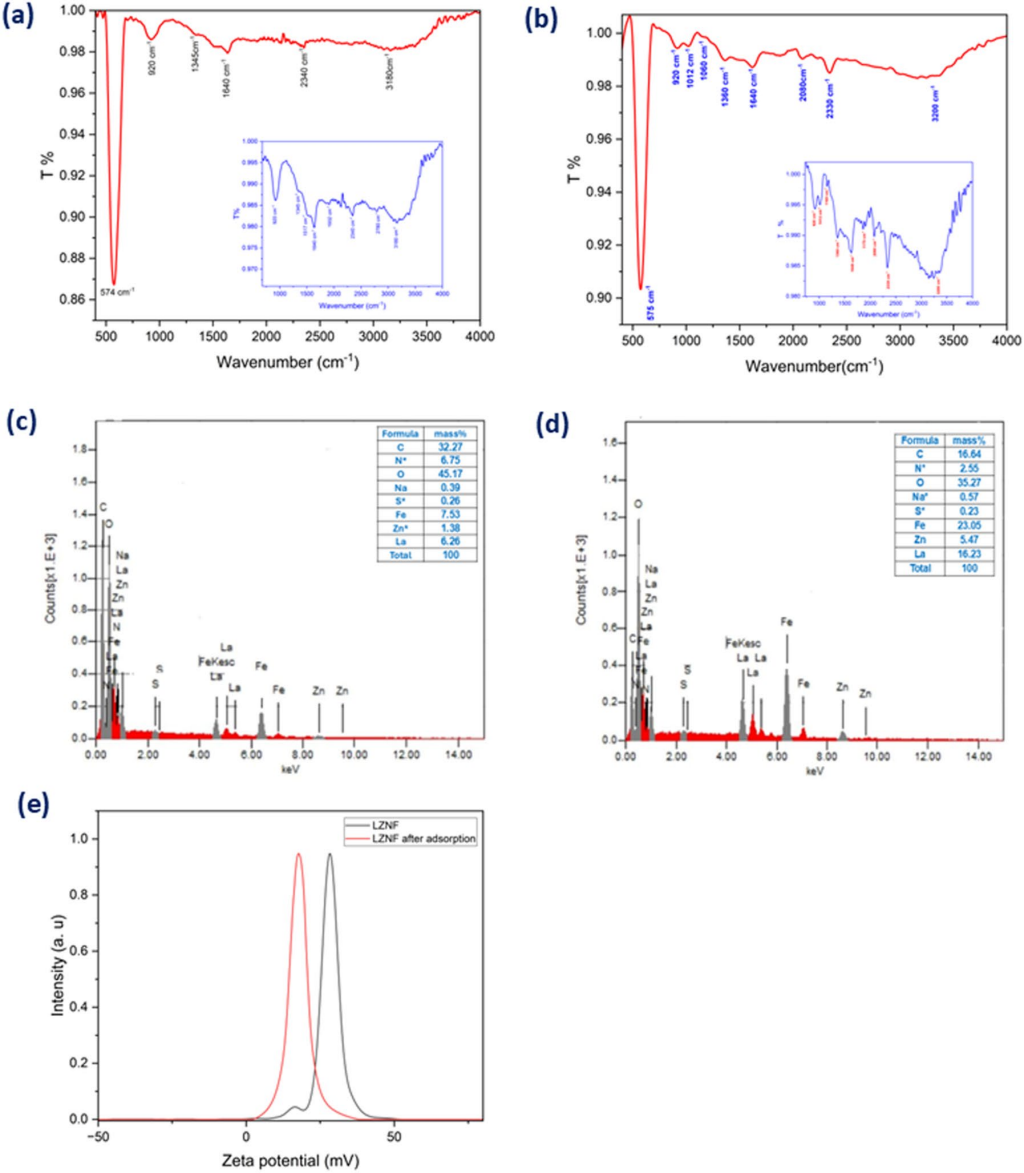
Characterization of adsorption, FTIR of LZNF(a) and LZNF/CS (b) after adsorption of IC, EDX for LZNF after adsorption of IC (c) and AB25(d), and Zeta potential of LZNF before and after adsorption of AB25.

A comparison of the FTIR spectra of LZNF and LZNF/CS before and after adsorption of IC dye (Fig. 15a and b, respectively) reveals several features that confirm successful adsorption. The broad band corresponding to O–H stretching, initially observed at around 3400 cm⁻¹ in both LZNF and LZNF/CS, shifts to a lower wavenumber (3200 cm⁻¹) and becomes broader after adsorption. This shift is attributed to hydrogen bonding between hydroxyl groups and the dye molecules^5^. Additionally, the band assigned to the metal–oxygen (M–O) bond at approximately 560 cm⁻¹ shifts to a higher wavenumber and becomes sharper, indicating perturbation of the metal–oxygen sites due to dye interaction^63^. Furthermore, Additional peaks associated with the sulfonate and amino groups of the IC dye were also detected in the spectra of dye-loaded LZNF and LZNF/CS, particularly at 920, 1022, 1640, 2080 and 2230 cm⁻¹, confirming successful adsorption^63^. The similarity between these newly appeared peaks and those characteristic of IC dye further supports the adsorption process. Overall, the adsorption of IC dye onto LZNF and the LZNF/CS nanocomposite is consistent with a physical adsorption mechanism dominated by hydrogen bonding and electrostatic interactions. Energy-dispersive X-ray (EDX) analysis of LZNF before and after adsorption of IC and AB 25 (Fig. 15c, d) further supports the dye uptake. After adsorption, new signals corresponding to the elemental constituents of IC and AB 25 (S, N, C, and Na) appear in the EDX spectra, in addition to the original LZNF elements (La, Zn, Fe, and O (Fig. 3a)), confirming the successful immobilisation of the dye molecules on the nanocomposite surface. The zeta potential of LZNF was measured before and after the adsorption of IC,  LZNF exhibited a highly positive zeta potential of about + 28 mV, consistent with a positively charged surface (Fig. 15e). After IC adsorption, the zeta potential decreased markedly to approximately + 17.8 mV, attributed to electrostatic attraction and binding of the dye’s anionic sulfonate groups (SO_3_^2−^) to positively charged surface sites, partially neutralising the surface charge.

### Proposed mechanism of dye adsorption

**Fig. 16:**
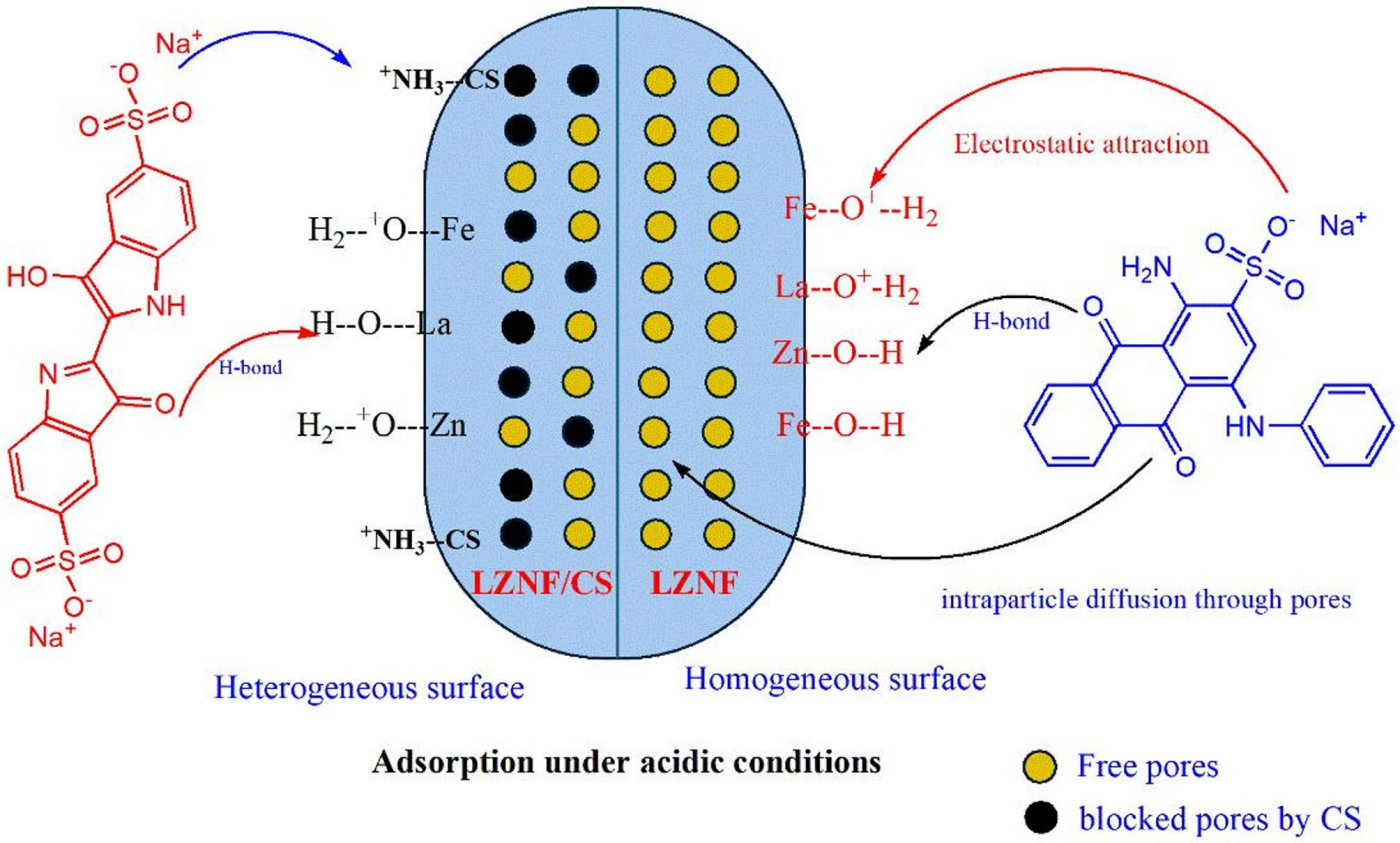
Proposed mechanism of the adsorption of AB25 and IC dyes on LZNF and LZNF/CS.

The adsorption mechanism of Indigo Carmine (IC) and Acid Blue 25 (AB 25) onto LZNF and its chitosan-modified nanocomposite (LZNF/CS) is primarily influenced by the solution pH, the point of zero charge (pH_pzc_) of the adsorbent, and the ionization properties (pKa) of the dyes. The pH of the solution alters the surface charge of the adsorbent by protonating or deprotonating functional groups, thereby modulating electrostatic interactions with dye molecules. LZNF has a pHpzc of 8.1, while Chitosan modification lowers this value to 5.9 due to the presence of hydroxyl (–OH) and amino (–NH₂) groups that readily protonate in acidic conditions. When the pH is below the pH_pzc_, the surface becomes positively charged, promoting electrostatic attraction to anionic dyes. Conversely, when the pH exceeds the pHpzc, the surface becomes negatively charged, repelling anionic species and potentially favouring interaction with cationic dyes. This behaviour aligns with the adsorption results for both IC and AB 25. The sulfonate groups (–SO₃⁻) in IC and AB 25 create strong electrostatic attractions with the positively charged LZNF surface at pH values below 8.1. Additionally, hydrogen bonding occurs between the –OH groups on the LZNF surface and the carbonyl and NH groups of the dyes. The introduction of Chitosan in LZNF/CS enhances adsorption under acidic conditions (pH 2), owing to the protonated –NH₃⁺ groups on the Chitosan backbone, which interact electrostatically with the anionic moieties of the dyes. Furthermore, the interaction of IC and AB 25 with the adsorbent likely involves hydrogen bonding between the carbonyl oxygen atom and the NH group in the dyes, as well as between the (–OH) and amino (–NH₂) groups on the Chitosan surface. The low adsorption enthalpies confirm that the process is predominantly driven by physisorption, with electrostatic attraction between protonated adsorbent groups and the anionic dye functionalities serving as the primary mechanism. At the same time, hydrogen bonding and π–π interactions act as secondary contributors (Fig. 16).

## Conclusion

In this study, La_0.5_Zn_0.5_Fe_2_O_4_/LaFeO_3_ (LZNF) nanoparticles were synthesized by coprecipitation, and La_0.5_Zn_0.5_Fe_2_O_4_/LaFeO_3_/CS (LZNF/CS) nanocomposites were prepared in situ using TPP as a crosslinker for low-molecular-weight Chitosan. The structure, surface characteristics, and composition of both nanocomposites were confirmed by EDX, FT-IR, VSM, XRD, TEM, SEM, Zeta potential, and N₂ adsorption–desorption analyses. XRD revealed that LZNF consists mainly of nanocrystalline cubic spinel La_0.5_Zn_0.5_Fe_2_O_4_, with minor orthorhombic LaFeO₃ as a secondary phase, with an average crystallite size of 10.25 nm. After in situ Chitosan polymerization, the crystallite size decreased to 8.25 nm, indicating that the Chitosan matrix can stabilise the nanoparticles and limit their growth. Both uncoated LZNF and chitosan-coated LZNF/CS exhibit S-shaped, nearly hysteresis-free magnetisation curves, consistent with superparamagnetic-like behaviour.

Textural analysis showed that LZNF and LZNF/CS possess specific surface areas of 72.21 and 31.65 m² g⁻¹, respectively, with average pore diameters of 5.33 and 3.10 nm, placing both materials in the mesoporous range. The higher surface area and more accessible pore structure of LZNF translate into superior adsorption performance in aqueous solution, achieving 97.7% and 92% removal of indigo carmine (IC) and Acid Blue 25 (AB 25), respectively, under acidic conditions, whereas LZNF/CS reaches 89% and 69% removal of IC and AB 25 at pH 2. These results indicate that, under the studied conditions, bare LZNF has a higher adsorption capacity and removal efficiency than its Chitosan-modified counterpart, which is attributed to the partial coverage and shielding of active ferrite sites by the Chitosan layer and the consequent reduction in total accessible surface area.

Adsorption kinetics for both dyes on LZNF and LZNF/CS follow a pseudo-second-order model, and the process is spontaneous and exothermic, confirming that chemisorption-type interactions dominate. Equilibrium data show that IC and AB 25 adsorption on LZNF is well described by the Langmuir model, consistent with monolayer coverage on a relatively homogeneous set of surface sites associated with the La-containing spinel/LaFeO₃ surface. In contrast, adsorption on LZNF/CS follows the Freundlich model, indicating heterogeneous and multilayer adsorption on a surface where Chitosan functional groups coexist with partially covered ferrite sites. For both adsorbents, electrostatic attraction between positively charged surface sites and the anionic sulfonate groups of the dye is the primary driving force, with hydrogen bonding and π–π stacking interactions providing additional contributions.

Overall, these results demonstrate that Chitosan modification does not enhance the maximum adsorption capacity relative to bare LZNF, owing to the reduced surface area and partial masking of highly active ferrite sites. Instead, it mainly alters surface chemistry and adsorption mode, so that LZNF provides higher capacity and removal efficiency for anionic dyes, whereas LZNF/CS offers a more heterogeneous adsorption interface and improved handling as a polymer-based nanocomposite.

## Data Availability

All data generated or analyzed during this study are included in this article.
